# Supplementary material for: Step-to-step variations in human running reveal how humans run without falling
Source: eLife. 2019 Mar 19;8:e38371. doi: 10.7554/eLife.38371 (PMC6424559; doi:10.7554/eLife.38371)
Supplement: Transparent reporting form [file elife-38371-transrepform.pdf]

eLIFE
1st Floor
24 Hills Road
Cambridge CB2 1JP, UK
P 01223 855340
W elifesciences.org
T @elife

eLife's transparent reporting form

Sample-size estimation

Our primary scientific questions involve data-based dynamical model building, not binary hypothesis testing. For such model-building, the relevant sample size number for the analyses performed here is the total number of strides run by the humans subjects, which is over 2075 strides across all subjects at a given speed, and about 260 per subject per speed. These sample sizes are sufficient for an expected error estimate in the regressed coefficients of less than 10% for a linear model with unit coefficients and large additive noise, corresponding to 20% R^2.

Replicates

Replication of entire trials is not relevant to the scientific questions being addressed here. Nevertheless, there is implicit replication, in that each running trial is many minutes long, and each block of a few running steps may be considered a replicate of another block. No steps or subjects were deemed outliers and no data points were rejected.

Statistical reporting

The relevant statistical analysis methods are described in the Materials and Methods section and where the results are reported. We also show a few scatter plots with hundreds of data points.

Group allocation

All human subjects performed all trials and were not separated into different experimental groups, as such group allocation is not relevant for the scientific questions we address here.

Additional data files ("source data")

All raw data have been posted on Dryad and linked to from the main article. The Dryad data link is: https://datadryad.org/review?doi=doi:10.5061/dryad.1nt24m0
and makes public 1 GB of anonymized human running data.

eLife Sciences Publications, Ltd is a limited liability non-profit non-stock corporation incorporated in the State of Delaware, USA, with company number 5030732, and is registered in the UK with company number FC030576 and branch number BR015634 at the address 1st Floor, 24 Hills Road, Cambridge CB2 1JP | August 2014
